# High-frequency oscillatory ventilation in adults: handle with care

**DOI:** 10.1186/s13054-014-0464-6

**Published:** 2014-08-29

**Authors:** Niall D Ferguson

**Affiliations:** Interdepartmental Division of Critical Care Medicine, Departments of Medicine and Physiology, and Institute of Health Policy, Management, and Evaluation, University of Toronto, Toronto General Hospital, 585 University Avenue, 11 PMB 120, Toronto, ON M5G 2N2 Canada; Department of Medicine, Division of Respirology, University Health Network and Mount Sinai Hospital, and the Toronto General Research Institute, 585 University Avenue, 11 PMB 120, Toronto, ON M5G 2N2 Canada

## Abstract

In the previous issue of *Critical Care*, Gu and colleagues reported the results of a systematic review and meta-analysis of randomized trials comparing high-frequency oscillatory ventilation (HFOV) with conventional ventilation in adults with acute respiratory distress syndrome (ARDS). In contrast to findings of prior meta-analyses, their main finding was that, despite reducing risks of oxygenation failure, HFOV does not improve survival in adults with ARDS.

In the previous issue of Critical Care, Gu and colleagues summarize the results of randomized controlled trials comparing high-frequency oscillatory ventilation (HFOV) to conventional ventilation in adults with acute respiratory distress syndrome (ARDS) in a meta-analysis [1]. In this report they incorporate results of new trials that were not available when the previous systematic review on the topic was published, which has led to a significant shift in conclusions [2].

HFOV emerged from physiological observations in the late 1970s and entered routine clinical practice in neonatal and pediatric ICUs in the 1980s [3]. In adult ICUs, HFOV uptake came at least a decade later, coincident both with technological advances that allowed oscillation of adult-sized patients and with increasing recognition of the clinical impact of ventilator-induced lung injury (VILI).

In contrast to conventional ventilation modes in which relatively large swings in airway pressure are used to generate tidal volumes, HFOV maintains a relatively constant mean airway pressure and produces small oscillations around this pressure to facilitate ventilation. These small pressure swings and resultant small tidal volumes are at the heart of the lung-protective potential of HFOV [4]. Because tidal volumes are so small (often less than anatomic dead space), the mean airway pressure in HFOV - which physiologically functions like positive end-expiratory pressure (PEEP) on conventional ventilation - can often be set much higher than usual PEEP settings because one does not have to leave room for a large increase in airway pressure with each inspiration. In this way, HFOV theoretically should be able to minimize VILI by limiting cyclic overstretching (volutrauma) while maintaining an adequate end-expiratory lung volume without opening and closing injury (atelectrauma) [5].

On this theoretical background, and with the support of animal studies showing enhanced lung protection with HFOV, clinical trials comparing HFOV with conventional ventilation in adults with ARDS have been conducted. This is the dataset that Gu and colleagues set out to summarize in their report. Using systematic search methods, they found six randomized trials including a total of 1,608 patients, although (it should be noted) 84% of these patients were enrolled in two trials published in 2013. The authors found no evidence supporting the use of HFOV in reducing 30-day (relative risk (RR) 1.05, 95% CI 0.83 to 1.36) or ICU (RR 1.22, 95% CI 0.93 to 1.64) mortality, and CIs were relatively wide in both cases. It is important to note that the authors found significant heterogeneity among the trials - perhaps not surprising given that they were conducted over the course of 15 years, with the evolution of ventilation practices observed over that time [6]. In this sense, it is difficult to interpret the promising results of the earlier trials since they used now-outdated control strategies with larger tidal volumes than would be prescribed today.

Significant heterogeneity also exists, however, even between the two recent trials - Oscillation for Acute Respiratory Distress Syndrome Treated Early (OSCILLATE) [7] and Oscillation in Acute Respiratory Distress Syndrome (OSCAR) [8] - which were designed, conducted, and published contemporaneously. OSCAR was designed as a pragmatic trial examining the introduction of HFOV to previously inexperienced ICUs and comparing this with usual care. In contrast, OSCILLATE was designed as a more explanatory trial, using centers with significant oscillation experience and employing strict ventilator protocols in both groups in an attempt to compare optimal open-lung HFOV with optimal open-lung conventional ventilation. OSCAR found identical outcomes between groups, although the usual-care control group received tidal volumes (mean day-1 tidal volume = 8.3 ± 2.9 mL/kg ideal body weight) that in some cases may have caused volutrauma. Meanwhile, OSCILLATE was stopped early for harm in the HFOV group, which may have been easier to detect given the comparison with strict tidal volume control and high levels of PEEP. Taken together (as shown by Gu and colleagues), these trials strongly suggest that there is no survival benefit to using HFOV as a primary mode for lung protection in most adults with moderate to severe ARDS. In this sense, this meta-analysis is important as it systematically incorporates these data into an updated estimate of treatment effect where previous reviews had suggested benefit of HFOV.

As the authors point out, none of the included trials specifically studied a common indication for HFOV in clinical practice: the patient failing conventional ventilation. In that sense, we cannot comment directly on this use of HFOV, although some may draw support for this indication from the improvements in oxygenation as documented here. While I believe that certain patients with severe ARDS may benefit from HFOV, the evidence compiled by Gu and colleagues suggests that these patients are uncommon and may be difficult to identify. Given the data supporting the use of other treatments in this subgroup, such as higher PEEP [9], neuromuscular blockade [10], and prone positioning [11], I believe that clinicians should reach for the oscillator in rescue settings only after having at least considered these other therapies.

## Abbreviations

ARDS: Acute respiratory distress syndrome
HFOV: High-frequency oscillatory ventilation
OSCAR: Oscillation in acute respiratory distress syndrome
OSCILLATE: Oscillation for acute respiratory distress syndrome treated early
PEEP: Positive end-expiratory pressure
RR: Relative risk
VILI: Ventilator-induced lung injury
